# Melatonin Prevents Chronic Kidney Disease-Induced Hypertension in Young Rat Treated with Adenine: Implications of Gut Microbiota-Derived Metabolites

**DOI:** 10.3390/antiox10081211

**Published:** 2021-07-28

**Authors:** Chien-Ning Hsu, Hung-Wei Yang, Chih-Yao Hou, Guo-Ping Chang-Chien, Sufan Lin, You-Lin Tain

**Affiliations:** 1 Department of Pharmacy, Kaohsiung Chang Gung Memorial Hospital, Kaohsiung 833, Taiwan; cnhsu@cgmh.org.tw; 2 School of Pharmacy, Kaohsiung Medical University, Kaohsiung 807, Taiwan; 3 Institute of Medical Science and Technology, National Sun Yat-sen University, Kaohsiung 804, Taiwan; howardyang@imst.nsysu.edu.tw; 4 Department of Seafood Science, National Kaohsiung University of Science and Technology, Kaohsiung 811, Taiwan; chihyaohou@webmail.nkmu.edu.tw; 5 Center for Environmental Toxin and Emerging-Contaminant Research, Cheng Shiu University, Kaohsiung 833, Taiwan; guoping@csu.edu.tw (G.-P.C.-C.); linsufan2003@csu.edu.tw (S.L.); 6 Super Micro Mass Research and Technology Center, Cheng Shiu University, Kaohsiung 833, Taiwan; 7 Department of Pediatrics, Kaohsiung Chang Gung Memorial Hospital and College of Medicine, Chang Gung University, Kaohsiung 833, Taiwan; 8 Institute for Translational Research in Biomedicine, Kaohsiung Chang Gung Memorial Hospital and Chang Gung University, College of Medicine, Kaohsiung 833, Taiwan

**Keywords:** asymmetric dimethylarginine, hypertension, chronic kidney disease, gut microbiota, melatonin, short-chain fatty acid, nitric oxide, trimethylamine-N-oxide, uremic toxin

## Abstract

Melatonin, a signaling hormone with pleiotropic biofunctions, has shown health benefits. Trimethylamine-N-oxide (TMAO) and asymmetric dimethylarginine (ADMA) are uremic toxins involved in the development of hypertension. TMAO originates from trimethylamine (TMA), a gut microbial product. ADMA is an endogenous nitric oxide (NO) synthase inhibitor. We examined whether melatonin therapy could prevent hypertension and kidney disease by mediating gut microbiota-derived metabolites and the NO pathway using an adenine-induced chronic kidney disease (CKD) young rat model. Six-week-old young Sprague Dawley rats of both sexes were fed a regular diet (C group), a diet supplemented with 0.5% adenine (CKD group), or adenine plus 0.01% melatonin in their drinking water (CKD + M group) for three weeks (N = 8/group). Adenine-fed rats developed renal dysfunction, hypertension, renal hypertrophy and increased uremic toxin levels of TMAO and ADMA. Melatonin therapy prevented hypertension in both sexes and attenuated kidney injury in males. Melatonin reversed the changes to the plasma TMAO-to-TMA ratio induced by CKD in both sexes. Besides, the protective effects of melatonin were associated with restoration of gut microbiota alterations, including increased α-diversity, and enhancement of the abundance of the phylum *Proteobacteria* and the genus *Roseburia* in male rats. Melatonin therapy also partially prevented the increases in ADMA in male CKD rats. Melatonin sex-specifically protected young rats against hypertension and kidney injury induced by CKD. The results of this study contribute toward a greater understanding of the interaction between melatonin, gut microbiota-derived metabolites, and the NO pathway that is behind CKD, which will help to prevent CKD-related disorders in children.

## 1. Introduction

Around 10% of the global population has chronic kidney disease (CKD) [1]. Adult kidney disease can originate in early life [2]. Through early identification and prevention of CKD from adulthood to childhood, we have the potential to slow or reduce the progression of CKD in later life. Accordingly, World Kidney Day 2016 declared the need to focus on kidney disease in childhood or even at an earlier stage [3]. Thus far, several animal models have been used to study CKD. Among them, rats fed a diet containing adenine have gained attention for producing most of the features that mimic human CKD [4]. However, little information currently exists with regard to the use of adenine-induced CKD in young rats to investigate pediatric CKD [5].

The gut microbiome and its derived metabolites have been shown to play roles in mediating CKD [6]. In CKD, gut microbiota dysbiosis causes the accumulation of gut-derived uremic toxins [7]. Trimethylamine-N-oxide (TMAO), a microbiota-derived uremic toxin, is related to increased cardiovascular events and mortality in the CKD population [8]. In the liver, TMAO originates from trimethylamine (TMA), a gut microbial product [9]. Prior research suggested that by increasing TMAO or TMA, high blood pressure (BP) could be induced in rats [9,10]. In a maternal adenine-induced CKD model, adult male offspring developed hypertension coinciding with abnormalities of the gut microbiota and a dysregulated TMA–TMAO pathway [11]. Besides, decreased short-chain fatty acid (SCFA) production has been related to increased BP [12].

Melatonin, N-acetyl-5-methoxytryptamine, is a biofunctional molecule widely distributed in nature [13]. Emerging evidence supports that early use of melatonin could be a potential strategy to prevent various chronic diseases in later life, including kidney disease [14]. It is known that melatonin, as well as its metabolites, act as antioxidants [15]. Nitric oxide (NO) deficiency is involved in the development of hypertension and kidney disease [16]. Asymmetric dimethylarginine (ADMA) can reduce NO production and induce oxidative stress [16]. In spontaneously hypertensive rats, melatonin has shown benefits against hypertension and oxidative stress via reduction of plasma ADMA and restoration of the NO pathway [17]. Although melatonin acts in a variety of ways to affect CKD [18], currently no information exists regarding its effect on gut microbiota-derived metabolites in pediatric CKD.

Accordingly, this research was carried out to elucidate whether melatonin therapy can protect young rats against CKD progression and hypertension and identify underlying protective mechanisms, focusing on the gut microbiota and its metabolites.

## 2. Materials and Methods

### 2.1. Animal Studies

All animal studies were approved by the Institutional Animal Ethics Committee (IACUC) of Chang Gung Memorial Hospital (permit number 2020031602). For the sample, 24 male and 24 female Sprague Dawley (SD) rats were purchased from BioLASCO Taiwan Co., Ltd. (Taipei, Taiwan). The rats were housed in an AAALAC-accredited facility. Food and water were available ad libitum. At six weeks of age, rats of both sexes received a regular diet (C group) or a diet supplemented with 0.5% adenine (0.25 mg/kg/day) for three weeks (CKD group). One group (CKD + M group) of the adenine-treated rats received 0.01% melatonin (10 mg/kg/day, Sigma-Aldrich, St. Louis, MO, USA) in their drinking water simultaneously. The dose was selected based on our previous studies in rats [19,20]. We used the CODA noninvasive BP system (a tail-cuff method, Kent Scientific Corporation, Torrington, CT, USA) to determine the rats’ BP at nine weeks old. Fresh fecal samples were collected, frozen, and stored at −80 °C until use. At nine weeks of age, the rats were anesthetized by intraperitoneally injecting ketamine (50 mg/kg body weight) and xylazine (10 mg/kg body weight) and were then euthanized by intraperitoneally injecting an overdose of pentobarbital for sacrifice. Heparinized blood samples were collected. The kidneys were subsequently collected. The plasma creatinine level was determined by high-performance liquid chromatography (HPLC) [12]. The animal care and experiments were conducted following established guidelines for the Care and Use of Laboratory Animals.

### 2.2. Analysis of TMA–TMAO Pathway

The plasma levels of TMAO, TMA, and their dimethylamine (DMA) metabolites were analyzed by liquid chromatography-mass spectrometry (LC-MS) using previously described methods [21]. For the LC-MS analysis, an Agilent 6410 Series Triple Quadrupole mass spectrometer (Agilent Technologies, Wilmington, DE, USA) with an electrospray ionization source was employed. We used diethylamine as an internal standard. Using an Agilent Technologies 1200 HPLC system, chromatographic separation was carried out on a SeQuant ZIC-HILIC column (150 × 2.1 mm, 5 μm; Merck KGaA, Darmstadt, Germany) protected by an Ascentis C18 column (2 cm × 4 mm, 5 μm; Merck KGaA). The eluate was monitored for DMA, TMA, and TMAO in multiple-reaction-monitoring mode using characteristic precursor-product ion transitions: *m*/*z* 46.1→30, *m*/*z* 60.1→44.1 and *m*/*z* 76.1→58.1, respectively.

### 2.3. Analysis of SCFAs

Fecal concentrations of acetate, propionate, and butyrate were determined by gas chromatography-mass spectrometry (7890B, Agilent Technologies Wilmington, DE, USA) equipped with an automated sampler, as we previously published [21]. We used the standard analytical grades of butyrate (from Chem Service, West Chester, PA, USA), acetate, and propionate (Sigma-Aldrich) as internal standards. We used a DB-FFAP column (30 cm × 0.25 mm, 0.25 µm; Agilent Technologies, Wilmington, DE, USA) for chromatographic separation. We used 2-ethylbutiric acid as the internal standard. An injection volume of 1 μL with a split ratio of 5:1 was performed at 240 °C. The fecal concentrations of SCFAs were adjusted by fecal weight and expressed as mM/g feces.

### 2.4. Gut Microbiota Compositions

Stool samples were analyzed with metagenomics focused on the V3-V4 of the 16S DNA gene using the methods published previously [21]. We used the Illumina MiSeq platform sequencing (Illumina, San Diego, CA, USA) and analyzed next-generation sequencing data at Biotools Co., Ltd. (Taipei, Taiwan). The sequences were clustered into operational taxonomic units (OTUs) using the USEARCH algorithm with a 97% sequence similarity threshold. Based on a representative sequence alignment with Fast-Tree, the phylogenetic relationships were constructed. We compared the diversity patterns of the microbial communities [22]. The alpha diversity was measured by Chao1 and the Shannon index [23]. We accessed the β-diversity of gut microbiota across groups using analysis of similarities (ANOSIM) and partial least squares discriminant analysis (PLS-DA) [24]. We used the linear discriminant analysis effect size (LEfSe) to discover high-dimensional biomarkers. The threshold of the logarithmic score (LDA) for discriminative features was set to four.

### 2.5. Analysis of NO Pathway

According to our validated protocol [21], high-performance liquid chromatography (HP series 1100; Agilent Technologies Inc., Santa Clara, CA, USA) with fluorescence detection of O-phthalaldehyde/3-mercaptopropionic acid (OPA/3MPA) derivatives was applied to determine the plasma levels of NO-related metabolites. These metabolites included L-citrulline (the precursor of L-arginine), L-arginine (substrate for NO synthesis), ADMA, and symmetric dimethylarginine (SDMA; inhibitors of NO synthase). Homoarginine (Sigma) was used as the internal standard.

### 2.6. Statistical Analysis

We used the Statistical Package for the Social Sciences software (SPSS Inc., Chicago, IL, USA) for analysis. All data are expressed as the mean ± the standard error of the mean (SEM). Comparisons within the three groups were analyzed by one-way analysis of variance (ANOVA) followed by Tukey’s post hoc test. A *p*-value of less than 0.05 was regarded as statistically significant.

## 3. Results

### 3.1. Blood Pressure and Renal Function

First, we observed that administration of adenine or melatonin did not induce death. The body weights (BWs) were lower in the CKD and CKD + M groups than the C group in both sexes (Table 1). In males, adenine administration caused a higher kidney weight and higher kidney weight-to-BW ratio in the CKD group vs. the C group, which was attenuated by melatonin therapy in males but not in females. At nine weeks of age, the systolic BP (SBP) and mean arterial pressure (MAP) were increased in the CKD group in males. Female rats receiving adenine also developed an increase in BP not only in SBP and MAP, but diastolic BP (DBP) as well, while the elevation of SBP in adenine-treated rats of both sexes was prevented by melatonin therapy. Additionally, Table 1 illustrates that adenine administration caused higher creatinine levels in the CKD groups of both sexes, which was partially prevented by melatonin therapy in males but not in females.

### 3.2. TMA–TMAO Pathway

Table 2 shows the plasma levels of TMA, TMAO, and DMA. In males, melatonin therapy increased the plasma TMA level in the CKD + M groups compared to the other two groups (both *p* < 0.001). The CKD and CKD + M groups had higher TMAO and DMA and a higher DMA-to-TMAO ratio in the plasma compared to the C group. Adenine administration caused a higher TMAO-to-TMA ratio in the CKD group (*p* < 0.001), which melatonin therapy prevented (*p* = 0.001).

Similar to males, adenine-treated female rats had higher TMAO and DMA and a higher TMAO-to-TMA ratio vs. the controls. However, the increased plasma TMAO-to-TMA ratio was prevented by melatonin therapy (*p* < 0.001). In addition, melatonin therapy caused the highest plasma DMA-to-TMAO ratio in the CKD + M group.

### 3.3. Fecal SCFA Levels

Table 3 illustrates that the changes in fecal SCFA concentrations were similar in both males and females. Adenine administration decreased the fecal concentrations of acetate, propionate, and butyrate in the CKD and CKD + M groups compared to the C group.

### 3.4. Gut Microbiota Compositions

**Figure 1:**
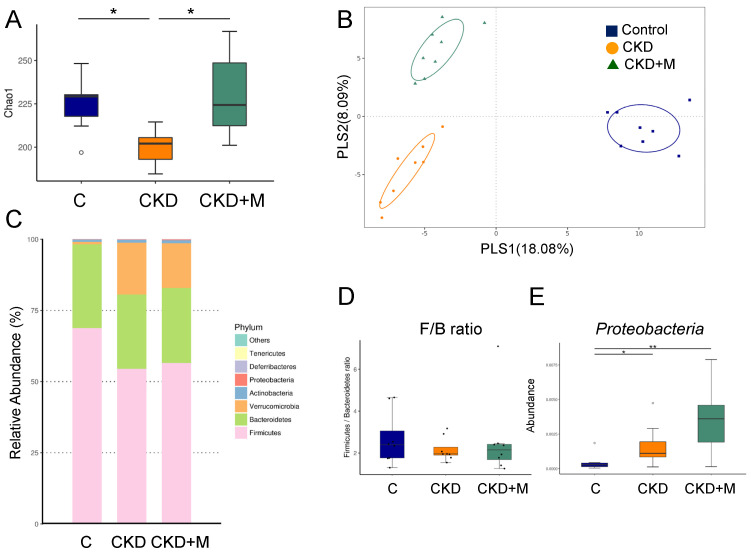
Gut microbiota composition in male rats. (**A**) α-diversity represented by Chao1 analysis, (**B**) β-diversity measured by partial least squares discriminant analysis (PLS-DA), (**C**) relative abundance of the top phyla of the gut microbiota among the three groups, (**D**) the *Firmicutes* to *Bacteroidetes* ratio, and (**E**) the abundance of phylum *Proteobacteria* among the three groups. N = 8/group. Data are shown as means ± SD. The single asterisk indicates *p* < 0.05 vs. C. The double asterisk indicates *p* < 0.01 vs. C.

We investigated how adenine and melatonin affected the gut microbiota composition. As shown in Figure 1A, the male CKD group displayed a lower α-diversity, represented by the Chao1 index, compared to the male C group (*p* = 0.033), which melatonin treatment prevented (*p* = 0.009). We next performed two β-diversity measures, the PLS-DA and ANOSIM, to compare the bacterial community similarity. Figure 1B illustrates scatterplots of the PLS-DA analysis with significant clustering according to the study group, showing that the gut microbiota structure of the C-group rats was distinctly altered by adenine and melatonin administration. The analysis of ANOSIM confirmed a significant difference in the gut microbiota among the three groups in males (all *p* < 0.05), indicating that the three groups had distinct enterotypes.

At the phylum level, the major bacteria phyla found were *Firmicutes*, *Bacteroidetes*, *Verrucomicrobia*, *Actinobacteria*, and *Proteobacteria* in male rats (Figure 1C). The *Firmicutes*/*Bacteroidetes* (F/B) ratio, a microbial marker related to BP [25], did not differ among the three groups (Figure 1D). The abundance of the phylum *Proteobacteria* was greater in the CKD and CKD + M groups than that in the C group (Figure 1E). At the family level, CKD significantly increased the abundance of *Deferribacteraceae* compared to the control (*p* = 0.003), which was prevented by melatonin therapy (*p* = 0.002).

**Figure 2:**
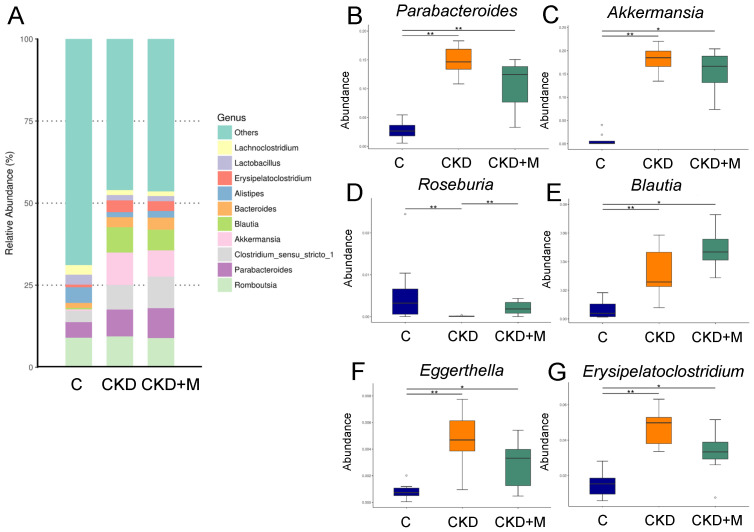
Gut microbiota at the genus level in male rats. (**A**) The relative abundance of the top 10 genera of the gut microbiota among the three groups. The abundance of genera (**B**) *Parabacteroides*, (**C**) *Akkermansia*, (**D**) *Roseburia*, (**E**) *Blautia*, (**F**) *Eggerthella*, and (**G**) *Erysipelatoclostridium* among the three groups. N = 8/group. Data are shown as means ± SD. The single asterisk indicates *p* < 0.05 vs. C. The double asterisk indicates *p* < 0.01 vs. C.

Figure 2A illustrates the relative abundance of major genera in the three groups in males. At the genus level, CKD significantly increased the abundance of the genera *Parabacteroides* and *Akkermansia* compared to the controls. These increases were not affected by melatonin therapy in the CKD + M group vs. the CKD group (Figure 2B,C). Conversely, CKD reduced the abundance of the genus *Roseburia*, which was prevented by melatonin therapy (Figure 2D). Additionally, the abundance of the genera *Blautia, Eggerthella*, and *Erysipelatoclostridium* were greater in the CKD and CKD + M groups vs. the control (Figure 2E–G).

**Figure 3:**
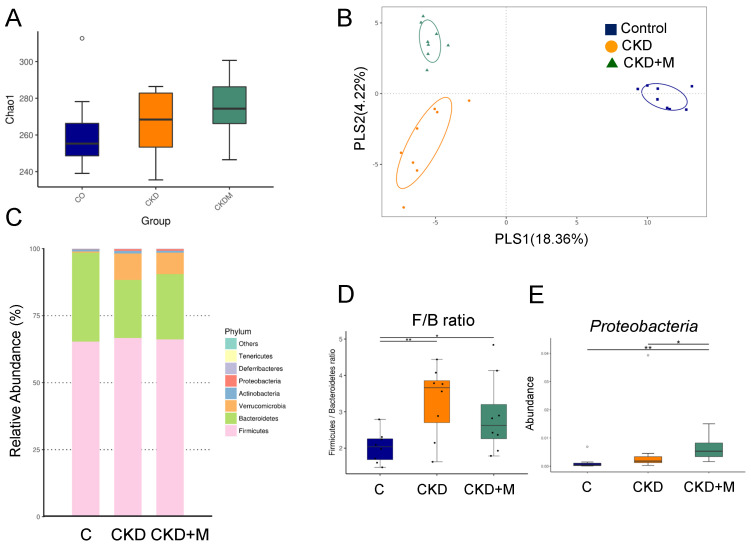
Gut microbiota composition in female rats. (**A**) α-diversity represented by Chao1 analysis, (**B**) β-diversity measured by partial least squares discriminant analysis (PLS-DA), (**C**) relative abundance of the top phyla of the gut microbiota among the three groups, (**D**) the *Firmicutes* to *Bacteroidetes* ratio, and (**E**) the abundance of the phylum *Proteobacteria* among the three groups. N = 8/group. Data are shown as means ± SD. The single asterisk indicates *p* < 0.05 vs. C. The double asterisk indicates *p* < 0.01 vs. C.

Unlike males, the α-diversity was not different among the three groups of females (Figure 3A). Figure 3B shows scatterplots of the PLS-DA analysis that are clearly separated, indicating the three groups displayed distinct enterotypes. Figure 3C shows the F/B ratio was higher in the female CKD and CKD + M groups than that in the C group (Figure 3D). The female CKD + M group showed a greater abundance of the phylum *Proteobacteria* than the other two groups (Figure 3E).

**Figure 4:**
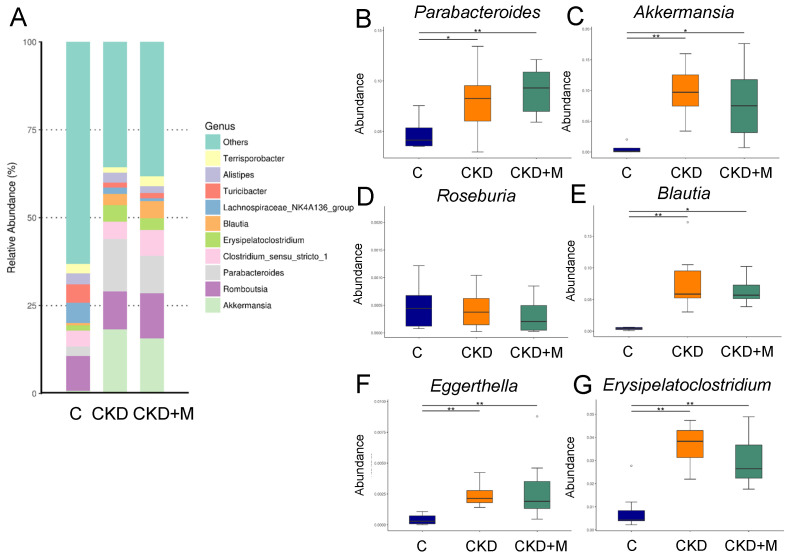
Gut microbiota at the genus level in female rats. (**A**) The relative abundance of the top 10 genera of the gut microbiota among the three groups. The abundance of genera (**B**) *Parabacteroides*, (**C**) *Akkermansia*, (**D**) *Roseburia*, (**E**) *Blautia*, (**F**) *Eggerthella*, and (**G**) *Erysipelatoclostridium* among the three groups. N = 8/group. Data are shown as means ± SD. The single asterisk indicates *p* < 0.05 vs. C. The double asterisk indicates *p* < 0.01 vs. C.

In females, the abundance of the genera *Parabacteroides*, *Akkermansia, Blautia, Eggerthella*, and *Erysipelatoclostridium* were higher in the CKD and CKD + M groups than the C group (Figure 4B,C,E–G). However, the abundance of the genus *Roseburia* did not differ among the three groups (Figure 4D).

**Figure 5:**
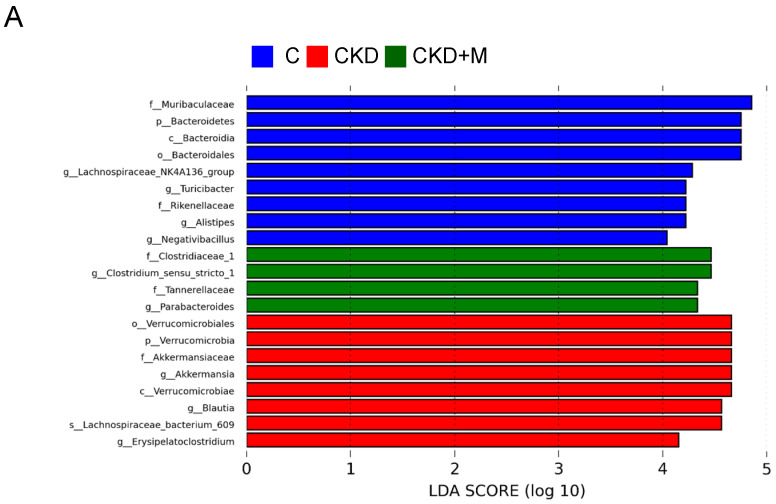

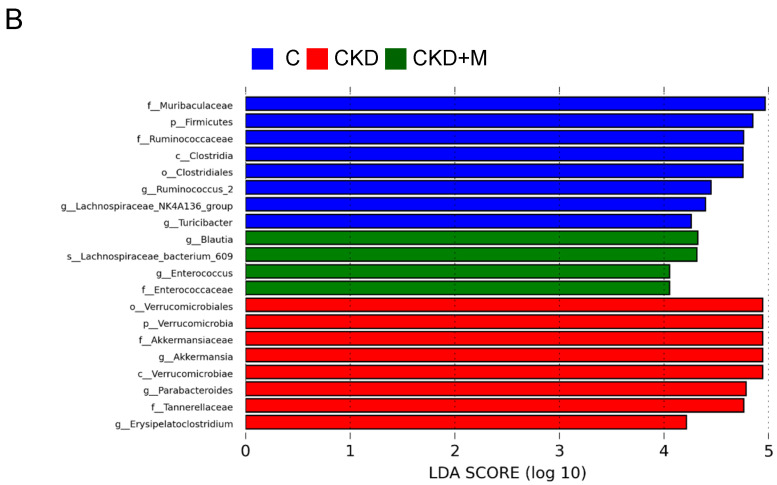
Linear discriminant analysis effect size (LEfSe) was applied to microbial marker discovery in metagenomic data. Here, are the most enriched and depleted bacterial taxa in (**A**) males and (**B**) females. C (blue) versus CKD (red) and CKD + M (green) are shown. Different taxonomic levels of bacteria are given, reaching from phylum down to the genus level. The threshold of the linear discriminant was set to four.

In Figure 5, the LEfSe algorithm identified several microbial markers that were significantly different between the groups. In the CKD group, the genera *Akkermansia* and *Erysipelatoclostridium* were in greater abundance in both sexes. Additionally, the genera *Blautia* in males and *Parabacteroides* in females were increased in the CKD group. Furthermore, the LEfSe analysis identified that melatonin significantly increased the abundance of the genus *Parabacteroides* in the male CKD + M group and the genera *Blautia* and *Enterococcus* in the female CKD + M group (Figure 5A,B).

### 3.5. NO-Related Parameters

We next analyzed NO-related parameters as hypertension and CKD are closely linked to NO deficiency [16]. As shown in Table 4, there was no difference in the plasma L-citrulline level among the three groups. The plasma ADMA level was higher, while the L-arginine-to-ADMA ratio was lower in the CKD group than the controls (both *p* < 0.05). In males, the plasma SDMA level was higher in the CKD and CKD + M groups vs. the controls. Melatonin therapy reduced the plasma L-arginine level and prevented increases in ADMA in CKD rats, while melatonin had a negligible effect on NO-related parameters in female CKD rats.

## 4. Discussion

The present study expands on earlier work regarding the health benefits of melatonin by showing that melatonin therapy prevents CKD-induced hypertension in both sexes and attenuates kidney injury in males; these results were achieved using an adenine-induced CKD model. We found it notable that the protective mechanisms of melatonin are related to the gut microbiota and TMA–TMAO pathway.

Our study clearly shows that adenine, when administered at the same dose and for the duration, is more nephrotoxic in young rats than in adult rats [4,12]. Similar to adults, adenine-treated young rats developed common features of human CKD, such as renal dysfunction, renal hypertrophy, hypertension, and increased uremic toxin levels. These findings suggest that young rats are more vulnerable than adult rats to adenine-induced kidney injury, in support of this model used to study pediatric CKD. In line with previous studies showing melatonin can treat or prevent hypertension in various models of hypertension [26,27], this is the first report of melatonin therapy preventing CKD-induced hypertension in a young rat adenine-induced CKD model. Additionally, melatonin showed beneficial effects against kidney injury in a sex-dependent manner, with melatonin mainly attenuating kidney injury in males but not females. Although melatonin has been shown to interact with sex hormones reciprocally [28], little information currently exists with regard to sex differences in terms of the therapeutic response of melatonin in CKD. Sex differences exist in CKD and women seem to be somewhat protected from developing end-stage renal disease [29]. However, this is in contrast to our findings. Thus, more attention should be focused on the sex-specific effect of melatonin, particularly in other CKD models. Further studies on the sex differences in the response to melatonin therapy may support the discovery of a new tool for the prevention of CKD.

In support of prior research showing that gut microbiota dysbiosis contributes to kidney disease and hypertension [6,12,30], adenine-induced CKD rats exhibited considerable alterations to the gut microbial composition. Consistent with previous studies reporting alterations of gut microbes in human and experimental CKD [25,30], young rats with CKD had a lesser abundance of beneficial microbes like *Roseburia*, but a greater abundance of the genera *Parabacteroides* and *Akkermansia*. Gut *Roseburia* spp. are part of the commensal bacteria with beneficial properties such as producing SCFAs, especially butyrate [31,32]. Our results also support a previous study showing that opportunistic pathogenic taxa like *Parabacteroides* were increased in a hypertensive gut microbiome, whereas SCFA producers such as *Roseburia* were decreased [33]. Moreover, consistent with our results, a previous study demonstrated that *Akkermansia* was increased, along with the progression of CKD [30]. Several studies have reported a potential association between a high F/B ratio and hypertension [11,25]. However, our study failed to identify a reduction in the F/B ratio in the CKD + M group, despite BP being reduced by melatonin therapy.

Consistent with previous studies demonstrating that uremic toxins are involved in CKD progression and hypertension [7,8,9], we observed that the elevated plasma level of TMAO coincided with hypertension and kidney dysfunction in adenine-induced young rats of both sexes with CKD. We observed that the plasma TMAO-to-TMA ratio was higher in the CKD group of both sexes, which melatonin prevented. Besides this, female CKD rats treated with melatonin had the highest plasma DMA-to-TMAO ratio among the three groups. The levels of plasma TMAO are determined by its renal excretion, its degradation to DMA, the TMA-to-TMAO conversion and TMA formation. A previous study showed a strong correlation between plasma TMAO levels and the glomerular filtration rate [34]. In CKD, increases in plasma TMAO can be mainly related to a decrease in the TMAO glomerular filtration. Besides this, TMAO can be metabolized to DMA or converted to TMA by TMAO reductase. Given that melatonin had a negligible effect on the renal function of female CKD rats, increased TMAO degradation, as reflected by the DMA-to-TMAO ratio, might be a protective mechanism behind CKD-induced hypertension. Another possible reason for increased TMAO might be increased TMAO-to-TMA conversion. Potentially, the TMAO-to-TMA ratio, as well as just the TMAO level, may be relevant to TMAO generation [35]. In accordance with the alteration to the TMA–TMAO pathway, alterations of the gut microbial community were observed. As *Deferribacteraceae* contributes to TMA production [36], similar to the TMAO-to-TMA ratio, male CKD rats had a higher abundance of *Deferribacteraceae*, which was prevented by melatonin therapy. Besides this, the phylum *Proteobacteria* was found to be the main contributor to the TMAO reductase pathway [37]. Melatonin therapy increased the TMA but decreased TMAO was associated with increases in the phylum *Proteobacteria*’s abundance, which supports the notion that augmenting the TMAO reductase pathway provides a protective mechanism.

In this study, we also examined other metabolites derived from the gut microbiota, SCFAs, which have been proposed to act as a mechanism linking the gut microbiota to BP control [38]. Although our findings in conjunction with others suggest that reduction of SCFAs may underlie hypertension associated with CKD [39], the protective effects of melatonin against hypertension seem not to be related to SCFAs and their producing bacteria. Moreover, prior studies involving patients with hypertension and healthy controls showed that *Blautia* occurred at a lower abundance in the hypertension group than in the control group, whereas *Eggerthella* and *Erysipelatoclostridium* were present at higher levels [40,41]. These observations are in support of our findings showing a low abundance of *Blautia* and a high abundance of the genera *Eggerthella* and *Erysipelatoclostridium* in CKD rats presenting hypertension.

Another possible mechanism of melatonin protecting male rats against CKD-induced hypertension and kidney injury may be, at least in part, related to a reduction in ADMA. Like TMAO, ADMA is a known uremic toxin [42]. Melatonin has been reported to protect against hypertension or kidney injury, coinciding with a reduction in an oxidative stress-induced increase in ADMA [43,44]. Melatonin therapy lowered BP, attenuated kidney injury, and reduced ADMA concurrently in male CKD rats, which were in agreement with the findings of previous studies [43,44].

Today, a broad spectrum of antihypertensive medication is accessible. However, the inadequate effectiveness and side effects of this medication lead patients with resistant hypertension to require invasive renal nerve denervation [45]. As a result, only a minority of these patients are satisfied with this therapy [45], not to mention its application in children. Thus, the search for a novel conservative treatment for resistant hypertension is unremitting. In children, CKD is the most common secondary cause of resistant hypertension. Melatonin is considered an essential candidate for the treatment of resistant hypertension [46] and our results show that melatonin even offers renoprotection beyond BP reduction, and therefore, has the potential future for clinical translation in the treatment of children with CKD.

Our study still has some limitations. One limitation is that we mainly analyzed gut microbiota and the NO pathway. Due to its pleiotropic biological activities, the protective effects of melatonin on hypertension and kidney injury might be attributed to other mechanisms. Another limitation is that we did not include a control + M group. The reason why we did not is that melatonin is currently used in humans and rats with a good safety profile [47]. However, it deserves further clarification as to whether the effects of melatonin therapy on young control rats differ from the effects on adult controls. Furthermore, we did not examine other dosages of melatonin; whether the physiological level of melatonin produces the same beneficial effects on CKD-induced hypertension awaits further evaluation. Last, the observations presented in our study are useful for indicating that melatonin has protective effects on CKD-induced hypertension by mediating gut microbiota-derived metabolites but are limited to testing in this model. Additional studies are required in other CKD models and in humans before melatonin can be translated into a clinical reality.

## 5. Conclusions

In this first adenine-induced pediatric CKD model, melatonin protected young rats from CKD-induced hypertension related to alterations of gut microbes involved in the TMA–TMAO pathway. The ADMA–NO pathway was also involved in CKD-induced hypertension. Providing a greater understanding of the alterations of gut microbiota and the derived metabolites that underlie CKD-induced hypertension, along with the beneficial effects of melatonin on protection from hypertension, our results can aid in preventing childhood hypertension and CKD progression.

## Figures and Tables

**Table 1:** Weights, blood pressure, and renal function.

Groups	C	CKD	CKD + M
Male	N = 8	N = 8	N = 8
Body weight (BW) (g)	360 ± 8	261 ± 11 *	237 ± 8 *
Left kidney weight (g)	1.78 ± 0.07	5.1 ± 0.44 *	4.09 ± 0.27 *#
Left kidney weight/100 g BW	0.49 ± 0.02	1.94 ± 0.12 *	1.73 ± 0.01 *#
Systolic blood pressure (mmHg)	128 ± 1	144 ± 1 *	132 ± 1 #
Diastolic blood pressure (mmHg)	86 ± 1	87 ± 1	87 ± 2
Mean arterial pressure (mmHg)	100 ± 1	106 ± 1 *	102 ± 1
Creatinine (μM)	22.7 ± 1	322.7 ± 25.7 *	235.9 ± 22.4 *#
Female	N = 8	N = 8	N = 8
Body weight (BW) (g)	244 ± 11	187 ± 6 *	174 ± 5 *
Left kidney weight (g)	1.12 ± 0.03	2.12 ± 0.12 *	1.91 ± 0.13 *
Left kidney weight/100 g BW	0.46 ± 0.02	1.14 ± 0.05 *	1.09 ± 0.05 *
Systolic blood pressure (mmHg)	118 ± 1	132 ± 1 *	121 ± 1 #
Diastolic blood pressure (mmHg)	75 ± 3	84 ± 3 *	81 ± 1 *
Mean arterial pressure (mmHg)	89 ± 2	100 ± 2 *	94 ± 1 #
Creatinine (μM)	23.4 ± 1.3	220.7 ± 23.5*	190 ± 18.2 *

* *p* < 0.05 vs. C; # *p* < 0.05 vs. CKD.

**Table 2:** Plasma levels of TMA, TMAO and DMA.

Groups	C	CKD	CKD + M
Male	N = 8	N = 8	N = 8
TMA (ng/mL)	455 ± 18	466 ± 26	786 ± 65 *#
TMAO (ng/mL)	301 ± 18	1427 ± 92 *	1376 ± 164 *
DMA (ng/mL)	111 ± 5	902 ± 95 *	809 ± 84 *
TMAO-to-TMA ratio	0.67 ± 0.05	3.13 ± 0.27 *	1.78 ± 0.19 *#
DMA-to-TMAO ratio	0.38 ± 0.02	0.63 ± 0.05 *	0.62 ± 0.07 *
Female	N = 8	N = 8	N = 8
TMA (ng/mL)	1653 ± 291	1877 ± 253	3543 ± 185 *#
TMAO (ng/mL)	424 ± 68	2037 ± 683 *	1453 ± 222 *
DMA (ng/mL)	202 ± 34	755 ± 51 *	931 ± 56 *
TMAO-to-TMA ratio	0.3 ± 0.05	1.13 ± 0.31 *	0.41 ± 0.06 #
DMA-to-TMAO ratio	0.47 ± 0.02	0.5 ± 0.06	0.7 ± 0.07 *

* *p* < 0.05 vs. C; # *p* < 0.05 vs. CKD.

**Table 3:** Fecal levels of acetate, propionate, and butyrate.

Groups	C	CKD	CKD + M
Male	N = 8	N = 8	N = 8
Acetate, mM/gm feces	7.68 ± 0.53	5.49 ± 0.6 *	5.25 ± 0.35 *
Propionate, mM/gm feces	2.99 ± 0.22	1.45 ± 0.44 *	1.49 ± 0.24 *
Butyrate, mM/gm feces	3.82 ± 0.66	1.15 ± 0.24 *	1.08 ± 0.15 *
Female	N = 8	N = 8	N = 8
Acetate, mM/gm feces	7.04 ± 0.95	3.93 ± 0.46 *	3.95 ± 0.35 *
Propionate, mM/gm feces	2.73 ± 0.35	0.91 ± 0.27 *	0.54 ± 0.09 *
Butyrate, mM/gm feces	5.1 ± 0.71	1.06 ± 0.14 *	0.7 ± 0.09 *

* *p* < 0.05 vs. C.

**Table 4:** Plasma levels of NO-related parameters.

Groups	C	CKD	CKD + M
Male	N = 8	N = 8	N = 8
L-citrulline (µM)	53.7 ± 1.6	47.6 ± 1.1	46.7 ± 1
L-arginine (µM)	165.8 ± 2.8	146.3 ± 4	118.8 ± 1.4 *
ADMA (µM)	1.65 ± 0.02	2.4 ± 0.04 *	1.96 ± 0.05 #
SDMA (µM)	1.21 ± 0.01	1.69 ± 0.04 *	1.61 ± 0.02 *
L-arginine-to-ADMA ratio (µM/µM)	102.4 ± 2.9	66 ± 1.9 *	58.8 ± 1.6 *
Female	N = 8	N = 8	N = 8
L-citrulline (µM)	56.1 ± 0.9	53.7 ± 0.8	55.8 ± 1.2
L-arginine (µM)	166.4 ± 2.5	160.9 ± 4.1	140.4 ± 3.2
ADMA (µM)	1.38 ± 0.03	2.1 ± 0.07 *	2.16 ± 0.05 *
SDMA (µM)	1.58 ± 0.07	2.13 ± 0.05	1.72 ± 0.02
L-arginine-to-ADMA ratio (µM/µM)	123.1 ± 3.4	83.2 ± 3.9 *	65.8 ± 1.2 *

* *p* < 0.05 vs. C; # *p* < 0.05 vs. CKD.

## Data Availability

Data is contained within the article.
